# ROBOTS IN SENIOR-LIVING FACILITIES: A SYSTEMATIC LITERATURE REVIEW

**DOI:** 10.1093/geroni/igac059.279

**Published:** 2022-12-20

**Authors:** Katie Trainum, Elliott Hauser, Bo Xie

**Affiliations:** The University of Texas at Austin, Austin, Texas, United States; University of Texas at Austin, Austin, Texas, United States; The University of Texas at Austin, Austin, Texas, United States

## Abstract

Robots are promising technologies for improving both the care of older adults living in facilities and the job satisfaction of caregivers, but concerns about their efficacy, ethics, and best practices remain. To inform this ongoing debate, we examined the literature on robots utilized in senior-living facilities. Three rounds of searching and screening were conducted in February 2022, following PRISMA guidelines. Keyword searches produced 666 non-duplicate articles from 5 relevant databases. We screened titles and abstracts against inclusion/exclusion criteria, resulting in 127 articles. Full-text screening resulted in 78 articles for analysis, published between 2002-2022 and represent 17 countries. Most studies demonstrated promising effects of robots, however there was a lack of high-quality studies, overrepresentation of female participants, and focus on companion robots over service robots. Few studies addressed the effects of robots on caregivers or controlled for novelty effects. Research addressing these gaps should combine robotic and gerontological expertise.

